# Assessment of the quality of life and mental health of healthcare students during the COVID-19 pandemic

**DOI:** 10.1590/0034-7167-2023-0068

**Published:** 2023-12-04

**Authors:** Pedro Henrique Batista de Freitas, Adriana Lúcia Meireles, Mery Natali Silva Abreu, Bruna Carolina Rafael Barbosa, Waléria de Paula, Clareci Silva Cardoso

**Affiliations:** I Universidade Federal de São João del-Rei. Divinópolis, Minas Gerais, Brazil; II Universidade Federal de Ouro Preto. Ouro Preto, Minas Gerais, Brazil; III Universidade Federal de Minas Gerais. Belo Horizonte, Minas Gerais, Brazil

**Keywords:** COVID-19, Quality of Life, Depression, Anxiety, Students, COVID-19, Calidad de Vida, Depresión, Ansiedad, Estudiantes, COVID-19, Qualidade de Vida, Depressão, Ansiedade, Estudantes

## Abstract

**Objective:**

to evaluate the quality of life (QOL) and the presence of symptoms related to depression, anxiety, and stress among students in the healthcare field, in comparison to the period before the COVID-19 pandemic.

**Methods:**

A comparative cross-sectional research was conducted at a Brazilian public university. QOL was assessed using the WHOQOL-bref scale, while symptoms of depression, anxiety, and stress were evaluated using the DASS-21 scale. Descriptive and inferential analyses were employed to compare the two time periods.

**Results:**

A total of 355 students participated in this study. During the pandemic, there were significant increases in severe depression symptoms (15.1% versus 24.8%), moderate anxiety (18.3% versus 29.4%), and moderate stress (40.9% versus 53.8%) observed among the participants. Additionally, a significant reduction in QOL was noted, particularly in the domain of social relationships (65.2 versus 59.6, p-value 0.029).

**Conclusion:**

The study highlights a deterioration in both the quality of life and the mental health of healthcare students during the COVID-19 pandemic.

## RESUMO

**Objetivo:**

Avaliar a qualidade de vida (QV) e a presença de sintomas de depressão, ansiedade e estresse em estudantes da área da saúde, em comparação ao período anterior à pandemia de COVID-19.

**Métodos:**

Pesquisa transversal comparativa em uma universidade pública brasileira. A QV foi avaliada por meio da escala *WHOQOL-bref*, enquanto os sintomas de depressão, ansiedade e estresse foram avaliados usando a escala DASS-21. Foram empregadas análises descritivas e inferenciais para comparar os dois períodos.

**Resultados:**

Um total de 355 estudantes participaram deste estudo. Durante a pandemia, foram observados aumentos significantes nos sintomas de depressão grave (15,1% versus 24,8%), ansiedade moderada (18,3% versus 29,4%) e estresse moderado (40,9% versus 53,8%) entre os participantes. Além disso, houve uma redução significativa na QV, especialmente no domínio das relações sociais (65,2 versus 59,6, p-valor 0,029).

**Conclusão:**

Evidenciou-se piora da QV e da saúde mental dos estudantes da saúde durante a pandemia de COVID-19.

## RESUMEN

**Objetivo:**

Evaluar la calidad de vida (CV) y la presencia de síntomas de depresión, ansiedad y estrés en estudiantes del área de la salud, en comparación con el período previo a la pandemia de COVID-19.

**Métodos:**

Investigación transversal comparativa realizada en una universidad pública brasileña. La CV se evaluó utilizando la escala WHOQOL-bref, mientras que los síntomas de depresión, ansiedad y estrés se evaluaron utilizando la escala DASS-21. Se emplearon análisis descriptivos e inferenciales para comparar los dos períodos.

**Resultados:**

Un total de 355 estudiantes participaron en este estudio. Durante la pandemia, se observaron aumentos significativos en los síntomas de depresión grave (15,1% versus 24,8%), ansiedad moderada (18,3% versus 29,4%) y estrés moderado (40,9% versus 53,8%) entre los participantes. Además, hubo una reducción significativa en la CV, especialmente en el ámbito de las relaciones sociales (65,2 versus 59,6, valor de p 0,029).

**Conclusión:**

Se evidenció un deterioro en tanto la calidad de vida como la salud mental de los estudiantes de salud durante la pandemia de COVID-19.

## INTRODUCTION

The COVID-19 pandemic, caused by the rapid spread of SARS-CoV-2, brought about unprecedented global transformations, resulting in high morbidity and mortality, along with significant social and economic impact, as well as a substantial decline in individuals’ quality of life (QoL)^(1)^. In this context, university students were profoundly affected by the pandemic, considering that the measures of social restriction, leading to lockdowns and the interruption of in-person education, significantly interfered with the routines and lifestyles of these students, indicating an impact on mental health and QoL^(2)^.

University students in the health sciences field have specific challenges throughout their academic training that make them more vulnerable to the development of mental health issues and a decrease in QoL^(3)^. These students encounter and face various stressors related to the academic environment during their training, such as an overload of activities, challenging experiences in clinical practice during internships, and pressure for results, particularly from public institutions^(4)^. Thus, there is some evidence pointing towards a deterioration of mental health and QoL during the COVID-19 pandemic, suggesting a high prevalence of depression, anxiety, and stress among health sciences students, with reports of affecting over 50% of this population^(5, 6)^.

QoL is a significant multidimensional indicator of health and the impacts of diseases and interventions, emerging as a metric capable of contributing to the understanding of pandemic outcomes^(7)^. In this perspective, it is crucial to investigate how the COVID-19 pandemic, especially during the most critical phase of lockdown, affected the QoL and mental health of students. This is due to the existence of gaps in this theme, as well as the understanding that potential deterioration in mental health can lead to reduced academic performance and unfavorable future consequences, such as worsening the quality of care provided to the population and increasing the risk of suicide within this group^(8)^.

## OBJECTIVE

To assess the quality of life and the presence of symptoms of depression, anxiety, and stress in health sciences students, compared to the period before the COVID-19 pandemic.

## METHODS

### Ethical Aspects

This investigation adhered to all guidelines and regulatory norms for research involving human subjects as outlined in Resolution 466/2012 of the National Health Council. It was approved by the Research Ethics Committee. All participants virtually read and agreed to the Informed Consent Form (ICF), which was presented prior to the questionnaire. A copy of the ICF was made available for download.

### Study Design, Period, and Location

This is a cross-sectional study guided by the STROBE (Strengthening the Reporting of Observational Studies in Epidemiology) tool, conducted with undergraduate students in the health sciences field at the Federal University of Ouro Preto (UFOP). Two epidemiological surveys were compared, one before and one during the COVID-19 pandemic.

This study is part of a multicenter epidemiological survey that investigated the prevalence of symptoms of depression and anxiety disorders among university students at federal institutions of higher education in the state of Minas Gerais, referred to as “PADu”^(4)^. The phase conducted during the critical context of the COVID-19 pandemic (lockdown) is part of the project “Effects of the COVID-19 Pandemic on Mental and Nutritional Health and the Home Food Environment of the Academic Community: Longitudinal Assessment – Padu COVID”^(9)^. UFOP is one of the oldest and largest universities in Brazil.

### Population and Inclusion and Exclusion Criteria

In this article, the study population consisted of students from four undergraduate courses in the health sciences field: Physical Education, Pharmacy, Medicine, and Nutrition. To represent the period before the pandemic, a subset of the original epidemiological survey data was used to form the final sample for that period, including data from UFOP students^(4)^. For the representative sample of the second phase, all students in the health sciences undergraduate courses of the same institution were invited via email to participate in the study. The sample included all those who responded to the self-administered questionnaire, considering the context of isolation imposed by the pandemic. Students who were on exchange during the data collection and those who provided repeated responses throughout the questionnaire items were excluded from the study. For each excluded student or non-respondent (refusal or no response), a replacement draw was conducted within the same profile.

### Study Protocol

Data was collected during the periods from May to December 2019 (before the pandemic) and from July 20 to August 27, 2020 (during the pandemic), both through virtual invitations. Students were invited to respond to the questionnaire via email and official university social media channels. The study was widely promoted on the university’s website. The questionnaire was made available on an online platform (Google Forms), and invitations were sent out biweekly in the first phase and weekly in the second phase.

The following variables were assessed:

• Sociodemographic and clinical
• Quality of Life measured by the WHOQOL-bref (World Health Organization Quality of Life scale – Bref)
• Mental health – symptoms of depression, anxiety, and stress evaluated using the Depression, Anxiety, and Stress Scale – DASS 21.

QoL was measured using the World Health Organization (WHO) WHOQOL-bref scale, duly adapted and validated for Brazil^(10)^. It is a generic instrument consisting of 26 questions, 2 of which are general (health and QoL), and the rest represent each of the 24 facets that make up the original instrument. The questions are divided into four domains: physical (7 questions), psychological (6 questions), social relationships (3 questions), and environment (8 questions). The scores of the individual domains are linearly scaled from 0 to 100 in a positive direction; in other words, the higher the score, the better the QoL in the last 15 days^(10)^.

The presence of symptoms of depression, anxiety, and stress was assessed using the DASS-21, also adapted and validated for Brazil^(11)^. It consists of 21 affirmative statements, subdivided into three subscales that self-assess symptoms of anxiety, depression, and stress over the past week. Each of these subscales consists of seven questions, and responses are obtained according to a 4-point Likert scale (0 to 3). The results of each subscale are obtained by summing the scores of its items and multiplying the total by two. Scores for depression, anxiety, and stress generated the following categories based on symptom severity: “normal”, “mild/moderate”, and “severe/very severe”.

### Results and Statistical Analysis

Descriptive and inferential analysis were used to compare variables before and during the critical period of the COVID-19 pandemic (lockdown). In descriptive analysis, absolute and relative frequencies were used, along with the calculation of measures of central tendency and dispersion. For the comparison of so-ciodemographic, clinical variables, and symptoms of depression, anxiety, and stress before and during, the Chi-Square and Fisher’s Exact tests were used. QoL scores were calculated following the syntax proposed by the WHO QoL group^(10)^, and the comparison of scores at the two moments was performed using the Mann-Whitney test. In comparisons, a *p* value ≤ 0.05 was considered significant. The software used for analysis was R (version 4.0.5).

## RESULTS

A total of 355 students participated in the study, with 93 in the first phase (before the pandemic) and 262 in the second phase (during the pandemic). Among these students, the majority were female (74.9%), with a mean age of 24 years, considering all participants. It was observed that the majority were single (93.2%), not engaged in paid work (84.6%), and resided in shared housing or boarding houses (55.7%). The comparison between the sociodemographic and clinical characteristics of the groups is described in Table 1.

**Table 1:** Comparison of sociodemographic and clinical characteristics of university health sciences students before and during the COVID-19 pandemic. Ouro Preto, Minas Gerais, Brazil, 2020 (N=355)

Variable	Before the pandemic n = 93 (%)	During the pandemic n = 262 (%)	p value
Courses			
Pharmacy	32.3	35.5	0.161**^II^**
Nutrition	21.5	30.5	
Medicine	29.0	22.5	
Physical Education	17.2	11.5	
Marital status			
Single	95.7	92.4	0.009^I^
Married	2.2	7.6	
Other^1^	2.2	0.0	
Biological sex			
Female	66.7	77.9	0.045^II^
Male	33.3	22.1	
Sexual orientation			
Heterosexual	82.8	81.3	0.243**^II^**
Homosexual	9.7	6.1	
Other^2^	7.5	12.6	
Paid work			
No	85.0	85.9	0.962**^II^**
Yes	15.1	14.1	
Housing			
Alone	8.6	11.5	0.085**^II^**
Parents/Relatives	25.8	36.3	
Other^3^	65.6	52.3	
Religion			
Catholic	48.4	49.6	0.976**^II^**
Evangelical	11.8	11.8	
Other^4^	39.8	38.6	
Alcohol consumption			
No	31.2	33.6	0.768**^II^**
Yes	68.8	66.4	
Excessive alcohol consumption			
No	38.2	48.6	0.168**^II^**
Yes^5^	61.8	51.5	
Cigarette/tobacco product use			
No	86.0	93.1	0.061**^II^**
Yes	14.0	6.9	
Illicit drug use			
No	55.9	92.0	**0.000^II^**
Yes	44.1	8.0	
Drug use initiated after university			
No	75.3	57.1	0.161**^II^**
Yes	24.7	42.9	
Intensification of drug use after university			
No	83.3	42.9	**0.000^II^**
Yes	16.7	57.1	
Physical exercise practice^6^			
No	43.0	24.1	**0.001^II^**
Yes	57.0	76.0	
Self-rated health			
Very Good	17.2	17.2	0.751^I^
Good	47.3	52.7	
Average	32.3	26.7	
Poor	3.2	3.4	
Chronic illnesses			
No	89.3	66.4	**0.000^II^**
Yes	10.8	33.6	
Family history of depression			
No	53.8	81.7	**0.000^II^**
Yes	46.2	18.3	
Family history of anxiety			
No	52.7	54.2	0.897**^II^**
Yes	47.3	45.8	
Age			
Mean (SD)	23.8 (3.5)	24.1 (4.8)	0.821^III^
BMI (Body Mass Index)^7^			
Mean (SD)	22.5 (3.7)	23.3 (3.8)	0.059^III^

*I Fisher's Exact Test; II Chi-Square Test; III Mann-Whitney Test; 1 Divorced, in a domestic partnership, other; 2 Bisexual, asexual; 3 Shared housing, boarding house, dormitory, spouse; 4 No religious affiliation, spiritualist, Eastern Buddhism, Jewish, Afro-Brazilian, other; 5 Total doses consumed on a single occasion (five doses for men and four for women, in a single occasion); 6 Engaging in physical activity refers to participating in physical exercise or any physical activity, such as sports (e.g., soccer, tennis, running, swimming, etc.); 7 BMI: Body Mass Index.*

In the comparison between the two time points (Table 1), a statistically significant difference was found regarding marital status, biological sex, use of illicit drugs, engagement in physical activity, and self-reported chronic illness (p < 0.05). There was an increase in the frequency of students who identified as married (2.2% versus 7.6%) and of female students (66.7% versus 77.9%). A significant decrease was also observed in those who reported using illicit drugs (44.1% versus 8.0%); however, those who reported intensifying their consumption after entering university showed an increase (16.7% versus 57.1%). Furthermore, there was an increase in physical activity (57.0% versus 76.0%, respectively) as well as in those who reported having some chronic illness (10.8% versus 33.6%).

The prevalence of anxiety, depression, and stress at the two time points is presented in Table 2. A statistically significant difference was observed between the two investigated time points regarding the severity of symptoms. Concerning depression symptoms, there was a slight increase in the frequency of mild/moderate symptoms (35.5% versus 38.6%) and an increase in severe/very severe symptoms (15.1% versus 24.8%), with a marginal difference between the two time points (p-value 0.052). Mild/moderate anxiety symptoms (18.3% versus 29.4%) increased significantly, while severe/very severe symptoms decreased (29.0% versus 19.9%), also showing a marginal difference (*p* value 0.054). A significant increase (*p* value 0.041) in the frequency of mild/moderate stress symptoms was observed (40.9% versus 53.8%), while severe/very severe symptoms remained almost unchanged at both time points (20.4% versus 20.6%).

**Table 2:** Prevalence of depression, anxiety, and stress symptoms among university health sciences students before and during the COVID-19 pandemic. Ouro Preto, Minas Gerais, Brazil, 2020 (N=355)

Variable	Before the pandemic n= 93 (%)	During the pandemic n=262 (%)	p value^1^
Depression			
Normal	49.5	36.6	0.052
Mild/Moderate	35.5	38.6	
Severe/Very Severe	15.1	24.8	
Anxiety			
Normal	52.7	50.8	0.054
Mild/Moderate	18.3	29.4	
Severe/Very Severe	29.0	19.9	
Stress			
Normal	38.7	25.6	0.041
Mild/Moderate	40.9	53.8	
Severe/Very Severe	20.4	20.6	

1 *Chi-Square Test*

Table 3 presents the comparison of QoL at the two time points, based on the domains of the WHOQOL-bref scale. A significant difference is observed (*p* value = 0.029) in the social relationships domain, as the score during the pandemic period was lower (65.2 versus 59.6). No significant differences were found between the groups in the other domains.

**Table 3:** Comparison of QoL of health university students, according to domains of the WHOQOL-bref scale, before and during the COVID-19 pandemic. Ouro Preto, Minas Gerais, Brazil, 2020 (N = 355)

Quality of Life Domains	n	Mean	Standard Deviation	p value^1^
Physical				
Before the pandemic	93	65.8	15.7	0.642
During the pandemic	262	65.1	16.2	
Psychological				
Before the pandemic	93	60.9	17.8	0.141
During the pandemic	262	57.8	19.2	
Social Relationships				
Before the pandemic	93	65.2	18.6	0.029
During the pandemic	262	59.6	19.8	
Environment				
Before the pandemic	93	63.9	14.9	0.270
During the pandemic	262	65.7	16.8	

1 *Teste de Mann Whitney; WHOQOL-bref - World Health Organization Quality of Life scale.*

## DISCUSSION

The results of this investigation point to an increase in symptoms of depression, anxiety, and stress among university students in the health field during the COVID-19 pandemic. Additionally, a reduction in Quality of Life (QoL) was evidenced, particularly in the domain of social relationships. Paradoxically, an increase in engagement in physical activity/sports and a decrease in the use of illicit drugs were observed.

A decrease in reports of illicit drug use among students during the pandemic was observed, as well as an increase in reports of intensified consumption after entering university within the same group. The prevalence of illicit substance use, alcohol, and tobacco is high among university health sciences students due to the pursuit of pleasure, social interaction needs, academic workload, and relief from anxiety and stress^(12)^. There are indications that in the initial phase of the pandemic, there was a decrease in the use of both legal and illegal drugs among these students, considering the influence of measures to contain the virus, such as social distancing and temporary interruption of academic activities^(13)^, leading many to return to living with parents and family rather than friends.

In this investigation, an increase in engagement in physical activities during the pandemic was observed. It is known that regular physical activity is associated with a positive impact on QoL and mental health, reducing stress and depressive symptoms among university health sciences students^(3, 14)^. With the imposition of lockdowns, a significant increase in sedentary behavior and physical inactivity was expected, which was observed in some studies^(^13,15^)^. However, the findings are not exclusive to this investigation, as the increase in physical activity in the early months of the pandemic was also observed in other research involving nursing and medical students^(16)^.

The reasons behind the increase in engagement in physical activities are not yet fully understood, nor is it clear whether this trend would persist if stricter isolation measures were maintained. It is believed that the student’s living environment influences their lifestyle habits, and the unique aspects of their health sciences education may have impacted their decision to exercise at home^(16)^. Additionally, during the first year of the pandemic, there were indications that individuals who regularly engaged in physical activities had a lower risk of unfavorable COVID-19 outcomes, such as hospitalization and death^(17)^, which may have also motivated students to continue or even increase their physical activity.

Depression and anxiety symptoms showed significant differences between the two time points in this investigation, with an increase in severe/very severe symptoms and mild/moderate symptoms, respectively, during the pandemic period. It is known that mental health problems, especially depression and anxiety disorders, are common among university health sciences students, with an increase in prevalence over the last decade and a connection to the academic environment and external factors^(18)^. Before the COVID-19 pandemic, the prevalence of depressive and anxiety symptoms, regardless of severity, was estimated to be over 30% among health sciences students^(4, 9, 19)^. There is evidence that the COVID-19 pandemic has had an impact on the mental health, social life, and education of these students, with the initial months being marked by significant changes in routine, including the interruption of classes, return to family homes, isolation, and uncertainty about their professional future^(20)^.

The increase in severe depression and moderate anxiety symptoms observed in this investigation indicates a possible influence of the pandemic period. A study conducted with American medical students during the early phase of the pandemic indicated a 70% increase in depression symptoms and a 61% increase in anxiety symptoms compared to studies conducted before the pandemic^(5)^. A meta-analysis involving nursing students found a prevalence of 52% for depression symptoms and 32% for anxiety symptoms, suggesting a relationship with the pandemic context^(6)^.

In Brazil, a multicenter study conducted with university students during the critical period of the pandemic indicated a prevalence of 60.5% for depressive symptoms and 52.5% for anxiety symptoms, with the main predictors being the presence of chronic illness, young age, and female sex^(2)^. These findings are similar to those of this study, which found a prevalence of 64.4% for depressive symptoms and 49.2% for anxiety symptoms, regardless of the level of severity. Other investigations also point to an increase in depression and anxiety symptoms among health sciences students, linked to social distancing, adaptation to the new routine, and a history of clinical and mental health issues^(21, 22)^.

There was a significant increase in mild to moderate stress symptoms, highlighting a concerning scenario during the initial months of the pandemic. Stress symptoms are common among students in the health field, displaying a complex interrelationship with anxiety and depression. Stress can be defined as a state of excessive, non-specific, excitement/tension resulting from the inefficiency or exhaustion of coping mechanisms, assessed by the severity of its occurrence^(11)^. In the context of the pandemic, a significant increase in stress symptoms, particularly moderate and severe ones, was observed, likely stemming from social distancing, adaptation to remote learning, and fear of contracting the virus^(23, 24)^.

In this investigation, it was found that 53.8% of students experienced mild to moderate stress symptoms during the pandemic. A multicenter study involving Brazilian university students also during the critical pandemic period found a similar result, identifying a prevalence of 57.5% for moderate stress symptoms using the same measurement tool^(2)^. These findings appear elevated when compared to a study assessing the presence of stress, anxiety, and depression symptoms in the Brazilian adult population during the pandemic, indicating a prevalence of 45.7% for stress symptoms.

Similarly, to depression and anxiety symptoms, studies suggest that the most relevant risk factors associated with the development of moderate and severe stress among university health sciences students during the COVID-19 pandemic include female sex, young age, sedentary lifestyle, financial difficulties, and a history of mental health issues^(22)^. There are indications that high levels of stress can impact the QoL of these students across all domains of the WHOQOL-bref scale, subsequently increasing the risk of depression and suicide attempts^(25)^.

Regarding QoL, a significant decrease was observed in the social relationships domain during the pandemic period. The social relationships domain encompasses an individual’s perception of their social support network, personal relationships, and sexual activity^(10)^. The COVID-19 pandemic drastically altered the routine and lifestyle of students, particularly during the first year, with a possible impact on QoL^(26)^. As such, there are indications that the QoL of university students was affected during the pandemic, with greater impairment in psychological and social relationship aspects, considering the confinement measures and the weakening of social and emotional bonds^(1, 7)^. The fact that data collection took place during the initial phase of the pandemic, in the period of lockdown, may have influenced the perception of QoL in the social relationships domain in this study.

A multicenter study conducted with Brazilian university students suggested a deterioration in QoL during the pandemic, notably concerning mental health, with the main predictors being young age, female sex, and a history of depression and anxiety disorders. Additionally, it indicated that health sciences students had lower QoL scores in the physical, psychological, and social relationships domains compared to students in other fields of study^(27)^.

In this context, it’s important to note that while the social relationships domain was the only one with statistical significance in this investigation, relatively low average QoL scores were observed in the other domains, particularly the psychological domain, during the pandemic. This finding may be related to the increase in depression, anxiety, and stress symptoms evidenced by this investigation, suggesting a potential impact of the pandemic period on the mental health and QoL of these students.

The potential significant increase in female students and those with chronic illnesses during the pandemic may have also contributed to the poorer perception of QoL and likely worsening of mental health, as suggested by this investigation. Prior to the pandemic, it was indicated that female students had a higher prevalence of mental disorders compared to male students, with evidence of poorer QoL, particularly in the psychological domain^(28)^. With the advent of the COVID-19 pandemic and its probable impacts, it is believed that female students may still have a higher prevalence of mental disorders and worse QoL, although longitudinal studies are needed to confirm this hypothesis^(13)^. Students with chronic illnesses also have worse QoL, being more vulnerable to the development of severe mental disorders^(29)^.

The findings of this investigation highlight a concerning scenario and the need for planning care strategies targeted towards health sciences students, given the various negative impacts of the COVID-19 pandemic on their health. It is suggested that these students experienced a significant impact on their QoL during the first year of the pandemic, with worsened mental health and social relationships, likely related to the high prevalence of mental disorders, especially depression, anxiety, and stress. Conversely, it is believed that students with good resilience and adequate social support have a better perception of QoL and, consequently, higher scores in the social relationships and psychological domains, primarily^(30)^.

### Study Limitations

This study has several limitations that are important to mention. Because a cross-sectional design was used, causal relationships between variables could not be established, mainly because the students who participated in the study were not the same across the two stages. Given that the samples were independent (first and second stages), the decision was made not to match them. Therefore, it is suggested to develop longitudinal and nationwide studies to better understand the impact of the pandemic on the mental health and QoL of these students.

### Contributions to the Nursing Field

This study revealed a challenging scenario in the context of education and health public policies for the studied population, considering the high burden of depression, anxiety, and stress symptoms found, as well as a possible decrease in QoL, particularly in the domain of social relationships. It is expected that the results will contribute to stimulating reflection and discussion processes within the academic community, focusing on the mental health of healthcare students, who are exposed to various vulnerabilities due to the nature of their education. Furthermore, by considering potential unknown future impacts of the COVID-19 pandemic, this study can aid in the development of health promotion policies and mental illness prevention, aiming to enhance the QoL of these future professionals and influencing the care they provide to the population.

## CONCLUSIONS

This investigation revealed a potential worsening of mental health and QoL among healthcare students during the COVID-19 pandemic, considering the significant increase in symptoms of depression, anxiety, and stress, along with a decrease in QoL scores in the social relationships domain. Therefore, given the current and future impacts resulting from the pandemic context, it is suggested that university administrators develop and implement strategies for identifying these mental health issues, providing referrals for treatment when necessary, and offering ongoing support throughout the course of their studies. Additionally, university administrators and the academic community should reflect on the planning of strategies that positively impact the QoL of these students, particularly related to healthy lifestyle habits and social support, aiming to enhance their resilience capacity.
